# Exercise or sports in midlife and healthy life expectancy: an ecological study in all prefectures in Japan

**DOI:** 10.1186/s12889-019-7570-y

**Published:** 2019-09-09

**Authors:** Takafumi Monma, Fumi Takeda, Haruko Noguchi, Hideto Takahashi, Taeko Watanabe, Nanako Tamiya

**Affiliations:** 1 Faculty of Health and Sport Sciences, University of Tsukuba, 1-1-1 Tennodai, Tsukuba-shi, Ibaraki 305-8574 Japan; 2 Advanced Research Initiative for Human High Performance, University of Tsukuba, 1-1-1 Tennodai, Tsukuba-shi, Ibaraki 305-8574 Japan; 3 Research and Development Center for Health Services, University of Tsukuba, 1-1-1 Tennodai, Tsukuba-shi, Ibaraki 305-8575 Japan; 4 Faculty of Political Science and Economics, Waseda University, 1-6-1 Nishi-Waseda, Shinjuku-ku, Tokyo, 169-8050 Japan; 5 National Institute of Public Health, 2-3-6 Minami, Wako-shi, Saitama 351-0197 Japan; 6 Faculty of Medicine, University of Tsukuba, 1-1-1 Tennodai, Tsukuba-shi, Ibaraki 305-8575 Japan

**Keywords:** Health inequality, Healthy life expectancy, Exercise, Sports, Middle-aged, Ecological study

## Abstract

**Background:**

With the increase of overall life expectancy in Japan, effective and beneficial lifestyle approaches and practices are crucial for individuals to have a long, productive and healthy life. Although previous studies suggest that exercise or sports, especially when performed with others, from midlife level have a positive impact on enhancing healthy life expectancy, there is paucity of information regarding these contexts and possible associations. The present study intends to clarify the relationship between engagement in exercise or sports among middle-aged persons and healthy life expectancy through an ecological study in all prefectures in Japan.

**Methods:**

We tabulated (1) the ratios of middle-aged individuals engaged in exercise or sports and (2) the different methods by which they are engaged in exercise or sports for each prefecture by using data from the 2005–2010 Longitudinal Survey of Middle-aged and Elderly Persons by the Ministry of Health, Labour and Welfare of Japan. Weighted multiple linear regression analyses were performed by sex, using healthy life expectancy in 2010 of each prefecture calculated by Hashimoto (2013) as a criterion variable; indices of (1) and (2) of each year as explanatory variables; and age, living conditions, employment, and chronic diseases as adjusted variables.

**Results:**

For middle-aged males, the ratio of those engaged in exercise or sports in each year from 2005 to 2010 was positively correlated with healthy life expectancy; this relationship was found in the ratio of middle-aged engaging in exercise or sports “with families or friends”. For females, such a relationship could only be found in the ratio of middle-aged females engaged in exercise or sports in 2008 and 2010, and those engaging in exercise or sports “with families or friends” in 2008.

**Conclusion:**

Prefectures with a higher ratio of middle-aged individuals engaging in exercise or sports, especially when done with families or friends, have longer healthy life expectancies. This was particularly evident for males. Thus, exercise or sports with families or friends in midlife seems to be more effective in promoting healthy life expectancy for males than females in Japan.

## Background

Although the Japanese have a long average lifespan, increased life expectancy does not necessarily indicate a higher quality of life. Longer life expectancy may increase the risk of diseases or disability before death [1, 2]. An older population also puts increasing pressure on the economy and social systems. Therefore, effective approaches for helping individuals to have a long, healthy life are necessary. The national health promotion movement in Japan, called “Healthy Japan 21 (the second term),” began in 2013 [3]. This campaign has two main targets: “extending healthy life expectancy” and “reducing health inequality.”

Healthy life expectancy is defined as the length of a person’s lifespan that is not characterized by limitations in daily activities due to health problems [3]. This is the measure that summarizes the information on one’s lifespan, health status and health-related quality of life [4, 5]. In Japan, healthy life expectancy is determined based on whether a person describes activity limitations in the Comprehensive Survey of Living Conditions (CSLC) [6], which is conducted by the Ministry of Health, Labour and Welfare (MHLW) of Japan. The difference between life expectancy and healthy life expectancy indicates the average number of years lived in poor health [5]. Therefore, it is necessary to extend healthy life expectancy rather than merely increase life expectancy.

The “health inequality” indicates the gap in healthy life expectancies among prefectures. Hashimoto calculated the healthy life expectancy in 2010 for each prefecture using data from the CSLC; the results of this study showed a gap of 2.79 years in males and 2.95 years in females among prefectures [7].

For the “Healthy Japan 21 (the second term)” campaign, physical activity and exercise are promoted as an approach to achieve these two main targets. Stenholm et al. [8] reported that leisure-time physical activity is associated with a greater increase in healthy life expectancy. Another study in Japan suggested that exercise among self-management groups may reduce the prevalence of disability among older adults [9].

Approaches to promote healthy practices are necessary not only for the elderly but also for middle-aged adults. Maintaining good health and avoiding unhealthy behaviors during the midlife period may reduce the risk of developing disabilities later in life [10]. Some studies demonstrate the benefits of exercise or sports on performing activities of daily living (ADL) among middle-aged adults. Ribeiro et al. [11] reported that leisure walking was important in maintaining ADL among these individuals. Our previous study, which used nationally representative data from the Longitudinal Survey of Middle-aged and Elderly Persons (LSMEP) [12] conducted by the MHLW of Japan, reported that exercise or sports, especially when performed with others, have a positive effect on the maintenance of ADL at 5-year follow-up, among middle-aged adults (50–59 years of age) [13].

Although these results seem to indicate a positive relationship between healthy life expectancy and exercise or sports, especially when performed with others, during the midlife period, this relationship has not been empirically verified. The present study intends to clarify the relationship between engagement in exercise or sports among middle-aged persons and healthy life expectancy through an ecological study in all prefectures in Japan.

## Methods

### Study population and procedures

The present study used data extracted from the LSMEP. Respondents to the survey were randomly extracted through stratified two-stage sampling. Firstly, 2515 districts were randomly selected from the complete set of 5280 districts surveyed in the 2004 CSLC. Secondly, 40,877 residents from each selected district were randomly chosen from the pool of individuals between 50 and 59 years of age in proportion to the district’s total population.

In 2005, the first year that the survey was conducted, questionnaires were dropped off at the respondents’ homes by enumerators who returned several days later to collect the self-reported surveys. From the second year onward, the questionnaire delivery method changed from “drop-off” to email, with respondents returning the questionnaire also by email. Questionnaires were only sent to respondents who had answered during the last year, the year before, or both years. The LSMEP did not recruit any new respondents since the first year of the survey’s implementation.

Data from the first through the sixth survey, i.e., from 2005 to 2010, were used in this study. Respondents included middle-aged individuals (50–59 years old in 2005 and 55–64 years old in 2010). The surveys included a total of 34,240, 32,285, 30,730, 29,605, 28,736, and 26,220 respondents, respectively (response rate: 83.8–97.3%). Among respondents returning their questionnaires, those who selected multiple options in the specific activity method for exercise or sports, those who had missing values for exercise or sports, and covariates were excluded from analyses. As a result, the final study sample in each group comprised 28,499, 22,223, 20,983, 22,281, 22,490, and 21,418 respondents, respectively (valid response rate: 68.3–83.2%).

### Study measurements

The LSMEP includes questions about exercise or sports. Respondents were asked if they had engaged in exercise or sports within the last year of the survey’s date. Those who had were further asked questions related to the specific activity methods of exercise or sports (“by oneself,” “with families or friends,” “with co-workers” [including former co-workers], “in a neighborhood community association,” or “in a non-profit organization or corporation in the public interest”). For the 47 prefectures investigated, the proportion of respondents was estimated for the following parameters: (1) engagement in exercise or sports and (2) specific activity methods of exercise or sports.

Other information retrieved from the LSMEP included age, sex, living arrangements (“living alone” or “living with others”), employment status (“employed” or “unemployed”), and chronic diseases (presence or absence of the following conditions: diabetes, heart disease, cerebral stroke, high blood pressure, hyperlipidemia, or cancer).

### Statistical analysis

We conducted weighted multiple linear regression analyses on the data. Some respondents dropped out of the LSMEP from the second year onward and during the subsequent follow-up. This phenomenon might be responsible for the differences in the attrition rate among the 47 prefectures surveyed. To attenuate the impact of attrition bias, we calculated a weight (W) using the following equation:

$$ {\mathrm{W}}_{\mathrm{n}}=\left({\mathrm{X}}_{\mathrm{n}}+{\mathrm{Y}}_{\mathrm{n}}\right)/{\mathrm{X}}_{\mathrm{n}} $$

n: Prefectures

X: No. of drop-out respondents

Y: No. of eligible respondents

We used healthy life expectancies from each of the 47 prefectures in 2010, which Hashimoto [7] calculated using the question regarding activity limitations in CSLC as a criterion variable; the rates of engagement in exercise or sports and of specific activity methods of exercise or sports for the 47 prefectures each year from 2005 to 2010 as explanatory variables; and age, living conditions (alone or otherwise), employment status (employed or not), and chronic diseases (presence or not) as adjusted variables.

## Results

The statuses of each variable from 2005 to 2010 are shown in Tables 1 and 2. The ratio of males engaging in exercise or sports was higher than that of females across all years; both the ratios increased over time (male average: 41.6–47.4%, female average: 38.9–45.4%). The proportion of respondents who reported doing exercise or sports “by oneself” and “with families or friends” was higher than other activity methods each year among both sexes. Males tended to exercise or sports “by oneself” more than “with families or friends,” whereas the exact opposite result was observed for females.

**Table 1:** Characteristics of middle-aged males in each prefecture between 2005 and 2010

	2005			2006			2007			2008			2009			2010		
	Mean ± SD	Min	Max	Mean ± SD	Min	Max	Mean ± SD	Min	Max	Mean ± SD	Min	Max	Mean ± SD	Min	Max	Mean ± SD	Min	Max
Age (Years)	54.6 ± 0.3	53.7	55.7	55.7 ± 0.2	55.1	56.6	56.7 ± 0.3	55.9	57.8	57.7 ± 0.3	57.1	58.6	58.7 ± 0.2	58.0	59.5	59.7 ± 0.3	59.2	60.7
Living with others (%)	93.5 ± 2.5	86.2	97.7	94.6 ± 2.3	87.4	98.7	95.0 ± 2.1	90.1	99.0	94.3 ± 2.2	89.1	97.5	94.4 ± 2.6	87.1	100.0	94.4 ± 2.5	86.8	98.4
Employed (%)	93.9 ± 1.7	89.6	97.6	92.6 ± 2.2	86.0	96.2	91.1 ± 2.7	83.5	96.4	89.1 ± 2.9	81.5	93.6	85.6 ± 2.8	79.5	90.7	83.7 ± 3.4	74.7	94.7
Presence of chronic diseases (%)	34.1 ± 3.5	24.3	41.1	46.2 ± 3.8	36.1	53.1	49.4 ± 3.9	37.6	58.6	48.5 ± 4.3	37.5	58.1	50.1 ± 4.1	41.1	58.7	50.4 ± 4.5	41.6	60.1
Exercise or sports (%)	41.6 ± 5.6	28.9	51.9	43.4 ± 6.0	32.3	57.0	44.7 ± 6.0	29.8	57.2	45.4 ± 6.2	31.8	58.6	46.5 ± 6.3	33.1	56.8	47.4 ± 6.1	34.2	57.6
Specific activity methods of exercise or sports																		
By oneself (%)	15.9 ± 2.9	11.0	22.7	17.4 ± 3.5	10.0	25.9	19.0 ± 3.5	11.9	26.5	20.2 ± 3.2	13.5	28.4	21.0 ± 3.5	10.9	28.1	22.5 ± 3.8	14.1	31.5
With families or friends (%)	15.0 ± 3.0	7.5	21.1	15.5 ± 3.8	7.2	25.2	15.5 ± 4.0	8.4	26.7	15.6 ± 3.8	5.4	24.1	16.2 ± 3.7	8.2	23.4	15.5 ± 4.1	6.4	26.5
With co-workers (%)	6.5 ± 2.0	2.1	10.6	6.4 ± 2.4	1.8	12.6	5.9 ± 2.3	2.0	10.5	5.3 ± 2.4	0.0	9.4	5.2 ± 1.6	2.4	8.6	4.6 ± 2.2	0.0	9.7
In a neighborhood community association (%)	3.5 ± 2.0	0.6	9.3	3.5 ± 2.6	0.0	11.3	3.6 ± 2.4	0.5	11.9	3.5 ± 2.5	0.0	12.0	3.4 ± 2.8	0.0	12.9	4.0 ± 3.3	0.8	20.0
In a non-profit organization or corporation in the public interest (%)	0.6 ± 0.6	0.0	2.7	0.7 ± 0.8	0.0	3.3	0.8 ± 0.9	0.0	3.6	0.7 ± 0.7	0.0	3.0	0.7 ± 0.6	0.0	2.4	0.8 ± 0.8	0.0	3.8

*SD* Standard deviation

**Table 2:** Characteristics of middle-aged females in each prefecture between 2005 and 2010

	2005			2006			2007			2008			2009			2010		
	Mean ± SD	Min	Max	Mean ± SD	Min	Max	Mean ± SD	Min	Max	Mean ± SD	Min	Max	Mean ± SD	Min	Max	Mean ± SD	Min	Max
Age (Years)	54.6 ± 0.2	54.0	55.0	55.6 ± 0.3	55.0	56.1	56.6 ± 0.3	55.8	57.1	57.6 ± 0.3	56.9	58.0	58.5 ± 0.3	57.9	59.1	59.6 ± 0.3	59.0	60.0
Living with others (%)	94.8 ± 2.3	89.7	100.0	94.5 ± 2.7	88.9	99.4	94.5 ± 2.5	88.4	98.9	94.1 ± 2.5	88.5	98.8	94.1 ± 2.2	89.2	97.9	93.7 ± 2.5	89.1	98.5
Employed (%)	70.4 ± 6.1	59.0	84.7	69.8 ± 6.0	59.5	81.6	68.1 ± 6.7	57.0	83.5	65.9 ± 5.6	55.2	78.4	62.4 ± 6.1	50.3	77.4	59.5 ± 6.6	46.7	72.8
The presence of chronic diseases (%)	27.7 ± 3.6	20.9	36.3	38.2 ± 3.4	31.3	46.1	43.2 ± 4.3	35.4	55.0	41.2 ± 3.9	30.4	51.7	42.3 ± 5.4	30.2	54.8	41.0 ± 4.1	32.8	49.7
Exercise or sports (%)	38.9 ± 5.9	27.1	49.9	42.0 ± 6.6	27.9	58.0	42.9 ± 6.9	25.7	54.5	44.4 ± 6.4	31.3	57.5	45.3 ± 7.2	33.3	57.4	45.4 ± 6.4	29.7	56.7
Specific activity methods of exercise or sports																		
By oneself (%)	15.6 ± 3.4	6.2	22.1	17.0 ± 3.9	7.8	26.1	18.6 ± 3.7	7.8	26.5	19.2 ± 3.6	10.2	26.0	19.8 ± 3.7	10.3	26.7	19.4 ± 3.9	10.4	27.9
With families or friends (%)	18.7 ± 3.7	11.5	26.9	20.4 ± 4.6	9.2	30.3	20.3 ± 4.3	10.0	29.0	20.7 ± 4.7	10.5	29.3	20.9 ± 5.1	11.8	33.3	20.5 ± 4.7	10.8	29.2
With co-workers (%)	1.6 ± 0.9	0.0	4.8	1.4 ± 1.0	0.0	4.4	1.1 ± 0.8	0.0	3.3	1.1 ± 0.8	0.0	3.1	1.1 ± 0.8	0.0	3.4	1.1 ± 0.8	0.0	2.9
In a neighborhood community association (%)	2.3 ± 1.3	0.0	6.2	2.3 ± 1.8	0.0	10.2	2.0 ± 1.2	0.0	5.0	2.6 ± 2.0	0.0	10.3	2.6 ± 1.7	1.0	8.6	3.3 ± 1.8	0.3	9.4
In a non-profit organization or corporation in the public interest (%)	0.7 ± 0.5	0.0	1.9	0.9 ± 0.7	0.0	2.8	0.9 ± 0.9	0.0	4.3	0.8 ± 0.7	0.0	2.3	0.9 ± 0.8	0.0	2.9	1.2 ± 0.8	0.0	3.2

*SD* Standard deviation

The results of the multiple linear regression analyses are shown in Tables 3 and 4. For males, the ratios of engagement in exercise or sports every year between 2005 and 2010 (β = 0.311–0.450), the ratios of engagement in exercise or sports “with families or sports” every year between 2005 and 2010 (β = 0.336–0.401), and the ratio of engagement in exercise or sports “in a neighborhood community association” in 2005 (β = 0.382) had a significantly positive relationship with healthy life expectancy in 2010. For females, the ratios of engagement in exercise or sports in 2008 (β = 0.356) and the ratios of engagement in exercise or sports “with families or friends” in 2006, 2008, and 2010 (β = 0.295–0.312) had a significantly positive relationship with healthy life expectancy in 2010.

**Table 3:** Relationships between healthy life expectancy in 2010 and exercise or sports among middle-aged males in each prefecture between 2005 and 2010

	2005		2006		2007		2008		2009		2010	
	β	*p*	β	*p*	β	*p*	β	*p*	β	*p*	β	*p*
Rate of Engagement in exercise or sports	0.333	0.029	0.311	0.031	0.313	0.049	0.400	0.006	0.450	0.002	0.402	0.004
Rate of specific activity methods of exercise or sports												
By oneself	0.000	0.999	0.075	0.606	0.106	0.478	0.182	0.211	0.205	0.182	0.240	0.105
With families or friends	0.349	0.026	0.379	0.031	0.401	0.019	0.369	0.013	0.342	0.019	0.336	0.022
With co-workers	−0.027	0.860	0.029	0.865	−0.207	0.166	−0.030	0.837	0.154	0.332	0.034	0.827
In a neighborhood community association	0.382	0.010	0.155	0.306	0.172	0.251	0.124	0.403	0.214	0.154	0.068	0.646
In a non-profit organization or corporation in the public interest	0.224	0.166	0.021	0.891	0.099	0.528	0.118	0.424	−0.042	0.798	0.001	0.992

Weighted multiple linear regression analysis adjusted for age, living arrangements, employment status, and chronic diseases

*N* = 47 (All prefectures in Japan)

**Table 4:** Relationships between healthy life expectancy in 2010 and exercise or sports among middle-aged females in each prefecture between 2005 and 2010

	2005		2006		2007		2008		2009		2010	
	β	*p*	β	*p*	β	*p*	β	*p*	β	*p*	β	*p*
Rate of Engagement in exercise or sports	0.258	0.064	0.247	0.060	0.046	0.750	0.356	0.019	0.150	0.282	0.295	0.067
Rate of specific activity methods of exercise or sports												
By oneself	0.240	0.076	0.083	0.528	0.001	0.995	0.121	0.431	0.097	0.488	0.221	0.167
With families or friends	0.163	0.289	0.302	0.025	0.138	0.348	0.312	0.032	0.116	0.406	0.295	0.050
With co-workers	−0.012	0.931	0.233	0.072	−0.179	0.233	0.107	0.465	− 0.038	0.775	− 0.131	0.397
In a neighborhood community association	0.173	0.230	−0.177	0.195	0.076	0.612	0.010	0.949	0.106	0.504	−0.210	0.158
In a non-profit organization or corporation in the public interest	−0.086	0.554	0.047	0.726	−0.280	0.077	0.063	0.663	0.021	0.880	0.018	0.904

Weighted multiple linear regression analysis adjusted for age, living arrangements, employment status, and chronic diseases

*N* = 47 (All prefectures in Japan)

## Discussion

Our study found a positive correlation between middle-aged persons’ engagement in exercise or sports and healthy life expectancy at the prefecture-level, specifically males. Prefectures with higher ratios of middle-aged males engaging in exercise or sports, specifically those engaging “with families or friends” between 2005 and 2010, had longer healthy life expectancies in 2010. Among females, only the ratios of engagement in exercise or sports in 2008 and that of engaging in exercise or sports “with families or friends” in 2006, 2008, and 2010 showed a positive correlation with healthy life expectancy. Hence, exercise or sports with families or friends seems more effective for prolonging healthy life expectancy in middle-aged males compared with middle-aged females.

The current finding aligns with those seen in a previous cohort study of Japanese individuals between 64 and 65 years of age which demonstrated exercise or sports can lower the risk of developing a disability among males, but not females [14]. Our results are also in agreement with the finding that a higher number of middle-aged males suffer from metabolic syndrome compared with those of females in the same age range [15]. Exercise or sports are effective preventative measures against developing metabolic syndrome and related conditions [16]. Furthermore, another study found that people are more likely to continue exercising or playing sports with companions than alone [17–19]. It has also been found that participation in sports reinforces social support [20]; therefore, participation with families or friends is thought to result in stronger social support for middle-aged people. Since social support is directly related to health indicators such as cancer and coronary heart disease [21], engagement in exercise or sports with families or friends may prolong healthy life expectancy.

Most Japanese have jobs with long working hours, and this may restrict the opportunities for middle-aged males to participate in exercise or sports. The national representative survey in Japan reported that the main reason for an interrupted exercise or sports was being busy in one’s job and daily life [22]. Government support and corporate efforts are essential for reducing overtime work hours. Fortunately, in 2018, the Japanese government enacted a labor reform bill that put a legal cap on overtime work. Additionally, the socioeconomic disparities in factors such as education, and income were associated with a lower likelihood of participation in regular exercise among Japanese adults aged < 60 years [23]. This finding may be due to the lack of appropriate skills and knowledge and having less time and money to devote to exercise or sports. Thus, enhancing health education and strengthening economic support may be required to reduce health inequality in areas with lower healthy life expectancy.

Meanwhile, musculoskeletal disorders, not cardiovascular diseases, are the main causative agent for activity limitation in females [24]. Exercise or sports could induce or exacerbate such disorders, and the rapid decrease in estrogen, bone mineral density [25, 26], and muscle mass [26, 27] occurs during menopause. Thus, exercise or sports may have a negative impact on activity in females. However, several reports suggest the vital role of exercise or sports in maintaining physical functionality among middle-aged females [13, 28, 29]. In other words, since exercise or sports may simultaneously be disadvantageous and advantageous to females, their association with healthy life expectancy is less clearly defined. Moreover, in terms of maintaining ADL among middle-aged people, females tend to enjoy taking up hobbies or learning in addition to exercise or sports, whereas males seem interested only in exercise or sports [13]. Therefore, these other activities may be sufficient for helping females prolong healthy life expectancy without participating in exercise or sports.

The present study has several limitations. Firstly, since it is an ecological study based on aggregated data, the possible bias caused by ecological fallacy cannot be excluded. Secondly, because participation in exercise or sports was assessed using a dichotomized scale, the amount of time spent on activities or a variety of them performed is unknown. Thirdly, the CSLC and the LSMEP were based on self-reported and retrospective questionnaires, which may entail recall and/or self-report bias.

Despite the aforementioned limitations, the present study also has several strengths. Firstly, the LSMEP focuses on a large and nationally representative sample of individuals, which mitigates selection bias. Our study also targeted a wide range of activity methods of exercise or sports, which was not done in previous studies. Finally, this study suggests effective approaches to reduce health inequalities among middle-aged adults, which has hardly been covered by previously published reports.

In conclusion, prefectures with a higher ratio of middle-aged individuals engaged in exercise or sports have longer healthy life expectancies. This is especially true among middle-aged males who participate in exercise or sports with their friends and family members. Hence, to prolong healthy life expectancy, it may be helpful to provide more support or a better environment for allowing middle-aged males to engage in exercise or sports with families or friends.

## Data Availability

All data from the LSMEP are collected by the MHLW in Japan and therefore, users of these data are strictly limited to those who obtain official permission from the MHLW, in accordance with Japanese Article 33 (Provision of Questionnaire Information) of the Statistics Act, by the Statistic Bureau, Ministry of Internal Affairs and Communications. Qualified researchers who would like to request access to the data should contact the Statistics and Information Department of the MHLW. Please refer to the following URL: http://www.mhlw.go.jp/toukei/sonota/chousahyo.html (In Japanese).

## Abbreviations

ADL: Activities of daily living
CSLC: Comprehensive Survey of Living Conditions
LSMEP: Longitudinal Survey of Middle-aged and Elderly Persons
MHLW: Ministry of Health, Labour and Welfare
